# Clinical applications of the venous excess ultrasound (VExUS) score: conceptual review and case series

**DOI:** 10.1186/s13089-021-00232-8

**Published:** 2021-06-19

**Authors:** Philippe Rola, Francisco Miralles-Aguiar, Eduardo Argaiz, William Beaubien-Souligny, Korbin Haycock, Timur Karimov, Vi Am Dinh, Rory Spiegel

**Affiliations:** 1 ICU Chief of Service, Santa Cabrini Hospital, Montreal, QC Canada; 2 Anasthesia & Surgery Critical Care Service, Hospital Universitario Puerta del Mar, Cádiz, Spain; 3 Instituto Nacional de Ciencias Medicas y Nutricion Salvador, Zubiran, Tlalpan, Mexico City, Mexico; 4 Division of Nephrology, Centre Hospitalier de L’Université de Montréal, Montreal, Canada; 5 Department of Emergency Medicine, Riverside University Health Systems Medical Center, Moreno Valley, CA USA; 6 Intensive Care, Hôpital Honoré Mercier, Ste-Hyacinthe, QC Canada; 7 Department of Emergency Medicine and Department of Internal Medicine, Division of Pulmonary and Critical Care Medicine, Loma Linda University Health, Loma Linda, CA USA; 8 Department of Critical Care, Georgetown University Medstar Washington Hospital Center, Washington, DC USA

**Keywords:** Point-of-care ultrasound, VExUS score, Congestive nephropathy, Venous congestion, Congestive heart failure

## Abstract

The importance of functional right ventricular failure and resultant splanchnic venous congestion has long been under-appreciated and is difficult to assess by traditional physical examination and standard diagnostic imaging. The recent development of the venous excess ultrasound score (VExUS) and growth of point-of-care ultrasound in the last decade has made for a potentially very useful clinical tool. We review the rationale for its use in several pathologies and illustrate with several clinical cases where VExUS was pivotal in clinical management.

## Background

The venous side of the circulation has long played second fiddle to the arterial side, who has enjoyed much of the medical fanfare both in acute and chronic disease. The arterial blood pressure, its tracing and its variation have been the subject of much study and are deeply ingrained in the day-to-day and even minute-to-minute of medical culture.

Of course, while arterial blood pressure is indeed critically important, the venous side may be just as critical as its more popular counterpart, and its physiological impact on organ function is vastly more important than commonly thought.

While the approach we discuss here is undoubtedly new, the physiology is not. As early as 1931, Winton noted that the impact of raising venous pressure was greater than an equal decrease in arterial pressure in terms of urine output [1].

**Fig. 1:**
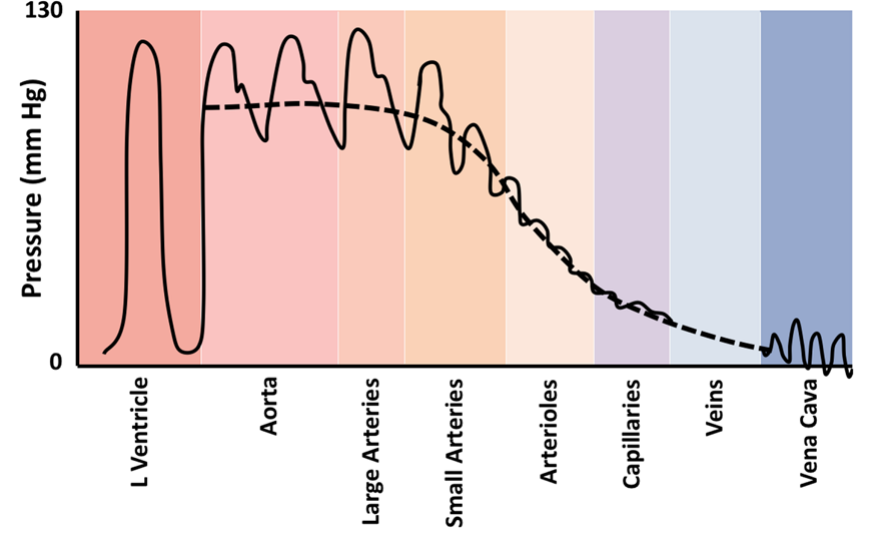
Pressure at different points in the circulatory system. Note the precapillary arteriolar pressure is substantially lower than the large arterial pressures

This is not entirely surprising when one realizes that the true perfusion pressure of an organ is in fact not mean arterial pressure (MAP) minus central venous pressure (CVP), but rather precapillary arteriolar pressure minus postcapillary venular pressure. In the former equation, the MAP is generally above 90, while in the latter, the inflow pressure can be in the 35–40 mmHg range (Fig. 1) [2]. Hence, the pressure gradient is much narrower, and the impact of raising venous pressure is much greater than commonly thought.

Ultrasound markers of venous congestion are not new either. Portal vein pulsatility has been described about 30 years ago in the setting of heart failure [3] although it was never incorporated in the comprehensive echocardiographic assessment.

The development of point-of-care ultrasound (POCUS) has brought together acute care clinicians and pathology with a powerful tool able to assess physiology at the bedside. The group of Denault and Beaubien-Souligny have been particularly instrumental in showing the strong link between portal vein pulsatility and acute kidney injury in the subgroup of cardiac surgical patients [4–6].

Recently, our group developed the venous excess ultrasound (VExUS) score, which incorporates hepatic venous, intrarenal venous Doppler and inferior vena cava (IVC) assessment to portal vein Doppler, and found the presence of a severe score to be very specific for the prediction of acute kidney injury following cardiac surgery, even more so than portal vein pulsatility alone [7].

In this case series, we will describe cases where the use of POCUS and more specifically, VExUS, was useful in identifying pathophysiology and assisting in clinical decision-making.

## Case presentations

### Case 1

A 75-year-old male was admitted with worsening dyspnea, anasarca and acute renal failure. The critical care outreach service was consulted for placement of a catheter to drain ascites for symptomatic relief. He was known for alcoholic cirrhosis and severe congestive heart failure with an ejection fraction of 20%. He had been getting frequent—almost weekly—paracentesis in the past 6 months to control his dyspnea. His admission creatinine was 121 mmol/L and rose in a sawtooth pattern over 2 weeks to 285 mmol/L, prompting alternating increases and decreases in his furosemide dose. In the next days, his renal function continued to deteriorate, furosemide was held and attempts were made to manage this cardiorenal syndrome using albumin infusions in an effort to improve a “low-flow state”. He was deemed to be in end-stage cardiorenal syndrome and the ascites management was meant to be a palliative therapy.

On examination the patient was in mild respiratory distress on nasal prongs, his blood pressure was 126/85, his heart rate 72 and his respiratory rate 24. His abdomen was distended and tense, he had marked anasarca involving both legs. His sensorium was clear, his extremities were warm and without mottling. There were chronic venous stasis changes in his lower legs.

**Fig. 2:**
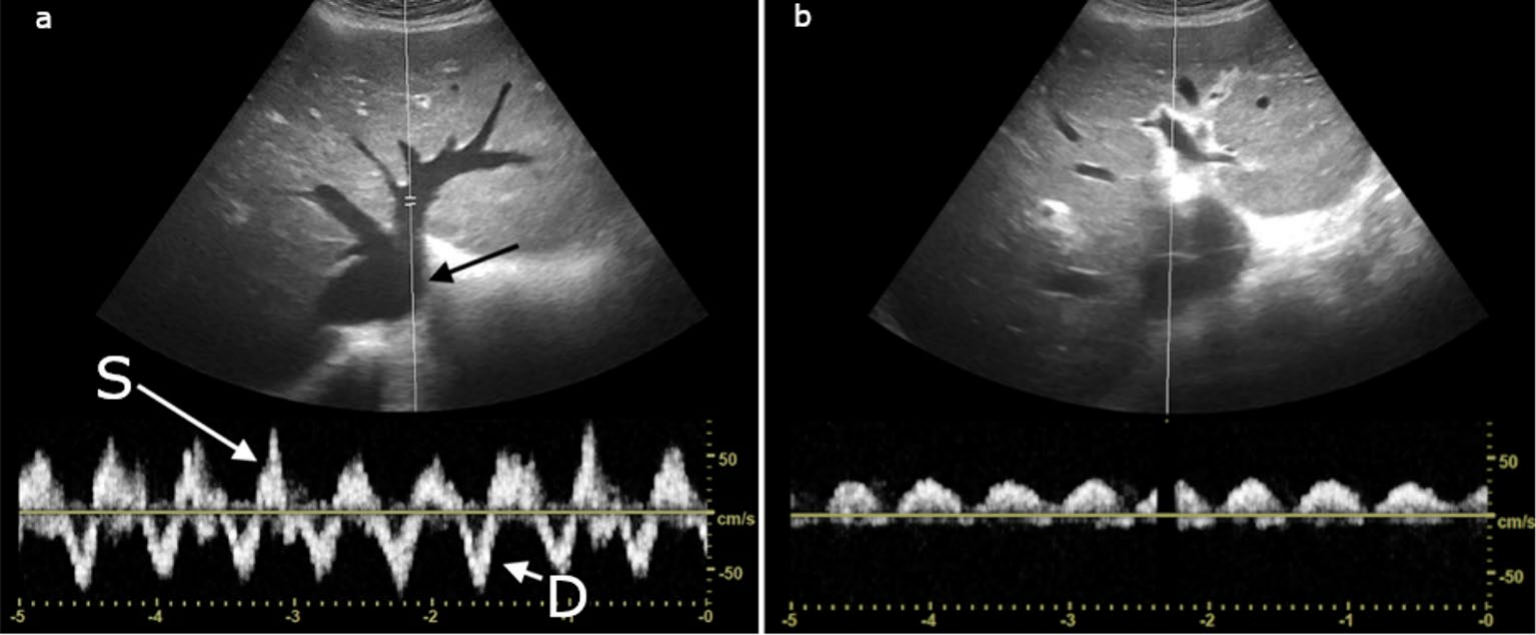
Case 1: **a** IVC short axis view (black arrow) and hepatic vein Doppler showing the D wave (short white arrow) and reversal of the S wave (long white arrow). **b** Portal vein Doppler showing > 100% pulsatility with some flow reversal

His POCUS examination revealed a large, dilated and fixed IVC measuring over 30 mm in both axes and along the whole intrahepatic course (Fig. 2a), his portal vein showed 100% pulsatility with reversal of flow (Fig. 2b), his hepatic vein Doppler showed reversal of flow of the S wave (Fig. 2a). These findings correspond to a VExUS grade 3 venous congestion. He had massive ascites and a severe dilated cardiomyopathy with an ejection fraction of 20%. He was admitted to the stepdown intensive care unit for drainage and further management.

Over the next days, continuous drainage of ascites was done until a total of 12 L had been removed. Intravenous furosemide was restarted at a higher dose until a net balance of negative 1000 cc per 8 h shift was obtained. Progressively, the pulsatility of the portal vein decreased until it was approximately 30%. The IVC regained respiratory variation and measured approximately 25–28 mm in both axes along most of its intrahepatic course. After an initial rise following admission to the intensive care unit (310 mmol/L/3.51 mg/dL), the creatinine decreased until it was normal (85 mmol/L/0.96 mg/dL).

The patient was discharged home with follow-up to the advanced CHF clinic.

In this case, the patient’s congestive cardiorenal syndrome was thought to be due to reduced effective circulating volume state due to poor left ventricular function, despite the lack of other clinical markers of poor perfusion. Essentially, a lack of forward flow was blamed for the renal failure, rather than poor perfusion due to congestive factors. This is a common error, and physical examination can be very challenging. The use of VExUS allowed the recognition of the venous congestive role in the patient’s problem. With aggressive fluid removal, the decongestion of the venous circulation via diuretics and ascites drainage allowed for improvement in renal perfusion pressure and function. It is important to note that while abdominal decompression may have helped by relieving direct pressure on the kidney, this alone did not resolve the portal vein pulsatility which remained significant after several liters having been drained, which instantly normalized intra-abdominal pressure clinically. Unfortunately this was not measured pre- and post-drainage, which would have been interesting.

This case also illustrates the difficulty of properly assessing venous congestion in the setting of cardiac and hepatic disease, as well as differentiating the sub-types of cardiorenal syndrome, and how POCUS with VExUS scoring can identify pathological congestion and guide therapy. The presence of a dilated IVC is not sufficient to demonstrate clinically important congestion, as the IVC may be chronically dilated with varying degrees of actual venous congestion. The commonly seen delay in changes in serum creatinine (as its serum value does not reflect renal function in real time) with interventions can make management challenging (whether attempting to increase or decrease intravascular volume), whereas with bedside physiological evidence of the presence or absence of venous congestion, more congruent therapeutic decisions may be made.

### Case 2

A 45-year-old male presented to the emergency department with worsening right upper quadrant abdominal pain after being referred for suspected acute cholecystitis diagnosed by ultrasound 2 weeks ago at another hospital. He arrived at the county emergency department with an ultrasound report indicating gallbladder wall edema and pericholecystic fluid, suspicious for acute cholecystitis. Because the report did not include images and was 2 weeks old, a repeat abdominal ultrasound and labs were ordered by an advanced care provider after consultation with the supervising physician.

The patient denied any past medical history and denied peripheral edema, dyspnea on exertion, or orthopnea. Vital signs were unremarkable and physical exam was significant for right upper abdominal tenderness to palpation, but no pedal edema, abnormal heart sounds, or crackles in the lung fields were appreciated in the noisy emergency department.

**Fig. 3:**
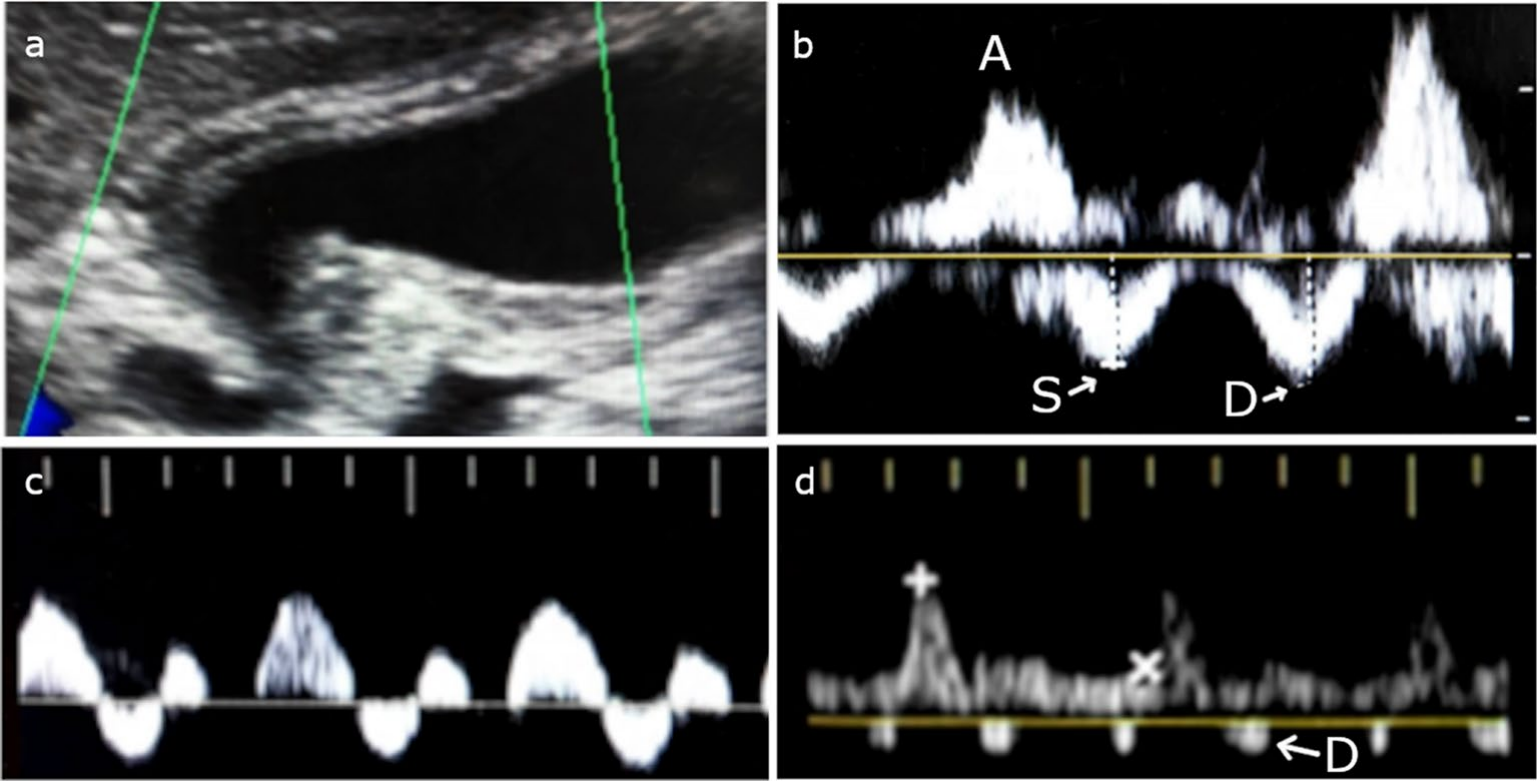
Case 2: **a **gallbladder showing signs of acalculous cholecystitis. **b** Hepatic vein Doppler with high amplitude A waves with S < D forward flow waves. **c** Triphasic portal vein Doppler **d** biphasic interrupted intrarenal venous flow

Laboratory results were significant for WBC of 14K, and mild elevations in AST and ALT. Creatine was 124 mmol/L (1.4 mg/dL). Ultrasound was interpreted by the radiologist as gallbladder wall thickening, pericholecystic fluid, without gallstones suspicious for acalculous cholecystitis (Fig. 3a). Surgical consultation was initiated by the advanced care provider and the surgery service evaluated the patient and planned to take him to surgery for a cholecystectomy later in the afternoon.

The supervising emergency physician was updated on the case and decided to review the ultrasound images. It was noted that the IVC and hepatic vein were plethoric and the portal vein Doppler was biphasic. Due to these findings, elevated venous pressure was suspected. A Focused Cardiac Ultrasound (FoCUS) and POCUS abdominal exam were therefore performed by the physician.

Abdominal POCUS was significant for a plethoric IVC without respiratory variation. Hepatic vein Doppler showed high amplitude A waves with S < D forward flow waves (Fig. 3b). There was a triphasic portal vein Doppler pattern (Fig. 3c), and biphasic interrupted intrarenal venous flow (Fig. 3d). These findings correspond to a VExUS grade 2 venous congestion, however there are severe flow abnormalities in the portal system. Furthermore, a more comprehensive bedside echocardiography exam showed biventricular dilation with a left ventricular ejection fraction of < 10%, restrictive left ventricular filling pattern, significant functional mitral regurgitation, and pulmonary hypertension. Left ventricular outflow tract velocity time integral (LVOT VTI) interrogation revealed a stroke volume of only 18 mL and cardiac output of 1.4 L/min.

The surgical service was contacted and after further discussion it was decided to cancel plans for surgery. The patient was discharged home for outpatient follow-up with cardiology and a furosemide prescription.

As shown in this case, the effects of venous congestion can mimic other pathological entities. In this particular instance, elevated venous pressures due to previously undiagnosed congestive heart failure led to impaired venous drainage of the portal system and congestion of the gallbladder wall which was initially mistaken as acute cholecystitis. When the ultrasound was reviewed, recognition of the plethoric IVC and abnormal Doppler findings of the portal vein combined with the lack of other findings suggestive of cholecystitis led to the both the diagnosis of congestive heart failure and prevented an unnecessary surgical procedure.

### Case 3

An 81-year-old male patient with moderate pulmonary hypertension and severe mitral insufficiency underwent mitral valve replacement with a bioprosthetic valve.

In the first 24 h, the patient needed transfusion of three units of blood, 800 mL of plasma and also pooled platelets. His fluid balance was positive by 2910 mL.

On physical examination, he weighed 90 kg, the heart rate was 73 bpm and arterial blood pressure was 138/56 mmHg. Oxygen saturation was 98% on high-flow nasal cannula (HFNC) with inspired oxygen at 40%. A pulmonary artery catheter showed moderate right ventricular (RV) dysfunction with pulmonary artery pressure 50/23 mmHg, central venous pressure (CVP) of 15 cm/H_2_O on noradrenaline 0.1 mcg/kg/min + dobutamine 10 mcg/kg/min. He was in mild respiratory distress and pulmonary auscultation revealed crackles in the lower third of both lung fields. In addition, there was mild edema in the lower limbs and pulses were normal. The hemoglobin was 9.5 mg/dL, creatinine 256 umol/L (2.9 mg/dL) and urea 18 (mmol/L 110 mg/dL).

**Fig. 4:**
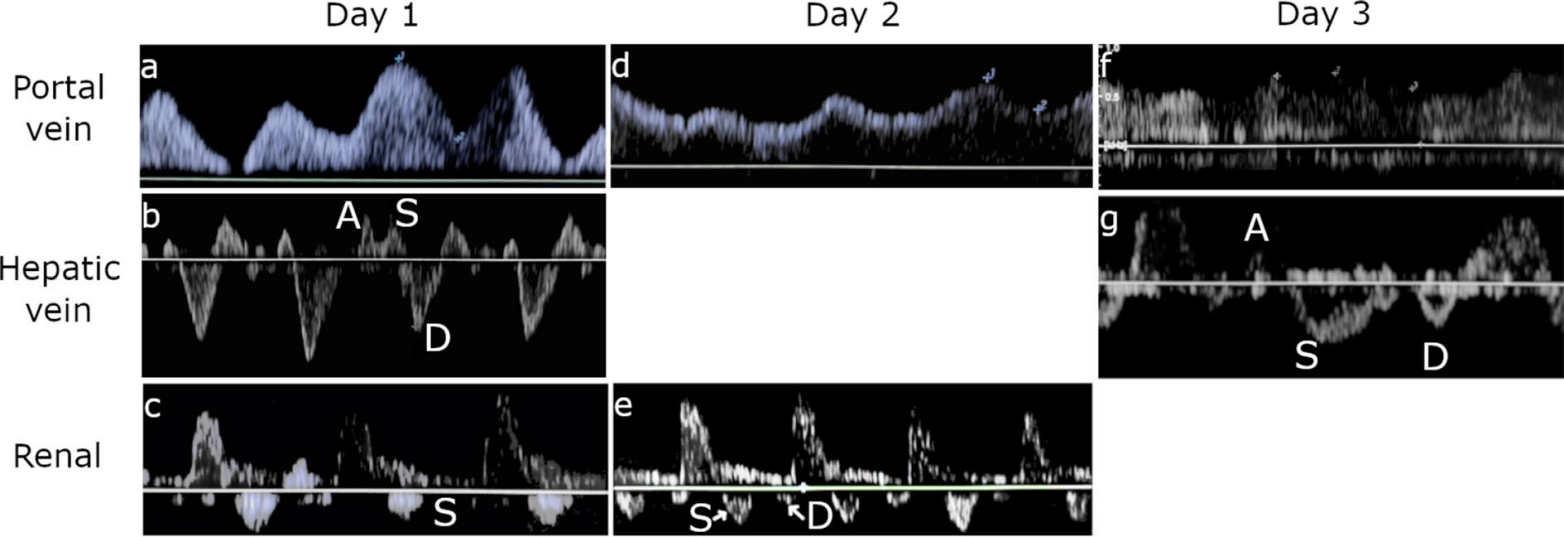
Case 3: Doppler profiles of portal vein, hepatic vein, and intrarenal vasculature at days 1, 2 and 3

His POCUS examination revealed an EF of 65% with impaired relaxation; moderate right ventricular dysfunction with tricuspid annular plane systolic excursion (TAPSE) of 13 mm. His VExUS score showed a dilated and fixed IVC measuring over 25 mm in both axes and his portal vein showed 65% pulsatility (Fig. 4a). Hepatic vein Doppler showed reversal of flow of the S wave (Fig. 4b) and renal Doppler showed discontinuous monophasic flow with only a diastolic phase (Fig. 4c). These findings correspond to a VExUS grade 3 venous congestion.

Intravenous furosemide was started at high dose until a net balance of negative 1200 cc per 24 h was obtained. The patient was then on noradrenaline 0.05 mcg/kg/min, dobutamine 5 mcg/kg/min with a HR of 87 bpm, BP of 127/45, PAP 48/19 mm/Hg, and a CVP of 10 mmHg. The Hb was 10.2 mg/dL.

Progressively, the pulsatility of the portal vein decreased until it was 34.7% (Fig. 4d) and renal Doppler improved to discontinuous biphasic flow with systolic and diastolic phases (Fig. 4e). The IVC regained respiratory variation and measured approximately 22 mm in both axes. After the initial rise, the creatinine decreased to 194 mmol/L (2.2 mg/dL) and the urea improved to 32 mmol/L (90 mg/dL).

On the third day, furosemide infusion was continued targeting a higher negative fluid balance. A negative balance of 3200 cc per 24 h was achieved and dobutamine was further decreased to 3 mcg/kg/min.

At this point the blood pressure was 130/70, heart rate 75 bpm, and CVP was 2 mmHg. POCUS examination showed an IVC measuring approximately 15 mm in both axes with a respiratory variation greater than 50%. The portal vein showed 20% pulsatility (Fig. 4f) and the hepatic vein Doppler improved to normal with S > D flow (Fig. 4g). Creatinine levels continued to decline to 132 mmol/L (1.5 mg/dL) and the urea to 21 mmol/L (60 mg/dL) and further improved to normal levels by the 4th day, and the patient was discharged to the cardiac surgery ward.

The pre-existence of pulmonary hypertension in this patient complicated the interpretation of the significance of the elevated CVP and dilated IVC. The chronicity of this condition leads to the question of the degree of previous compensated congestion versus the contribution of more acute elevations in venous pressures. VExUS allowed the assessment of venous congestion and its contribution to the patient’s hemodynamic derangement. Despite inotropic support, the RV was significantly volume overloaded leading to decreased venous return in the face of acutely elevated right atrial pressures. This in turn led to a decreased perfusion gradient of the kidneys and likely further renal fluid retention. Diuresis thus allowed venous decongestion and improved renal perfusion in addition to offloading the RV, returning it to the steeper part of the Frank–Starling curve.

### Case 4

A 57-year-old patient with group 1 pulmonary hypertension had been recently seen at an ambulatory clinic with an increase in the serum creatinine to 235 mmol/L (2.66 mg/dL) (baseline 96 mmol/L/1.09 mg/dL). Because of this, bumetanide and spironolactone were suspended.

**Fig. 5:**
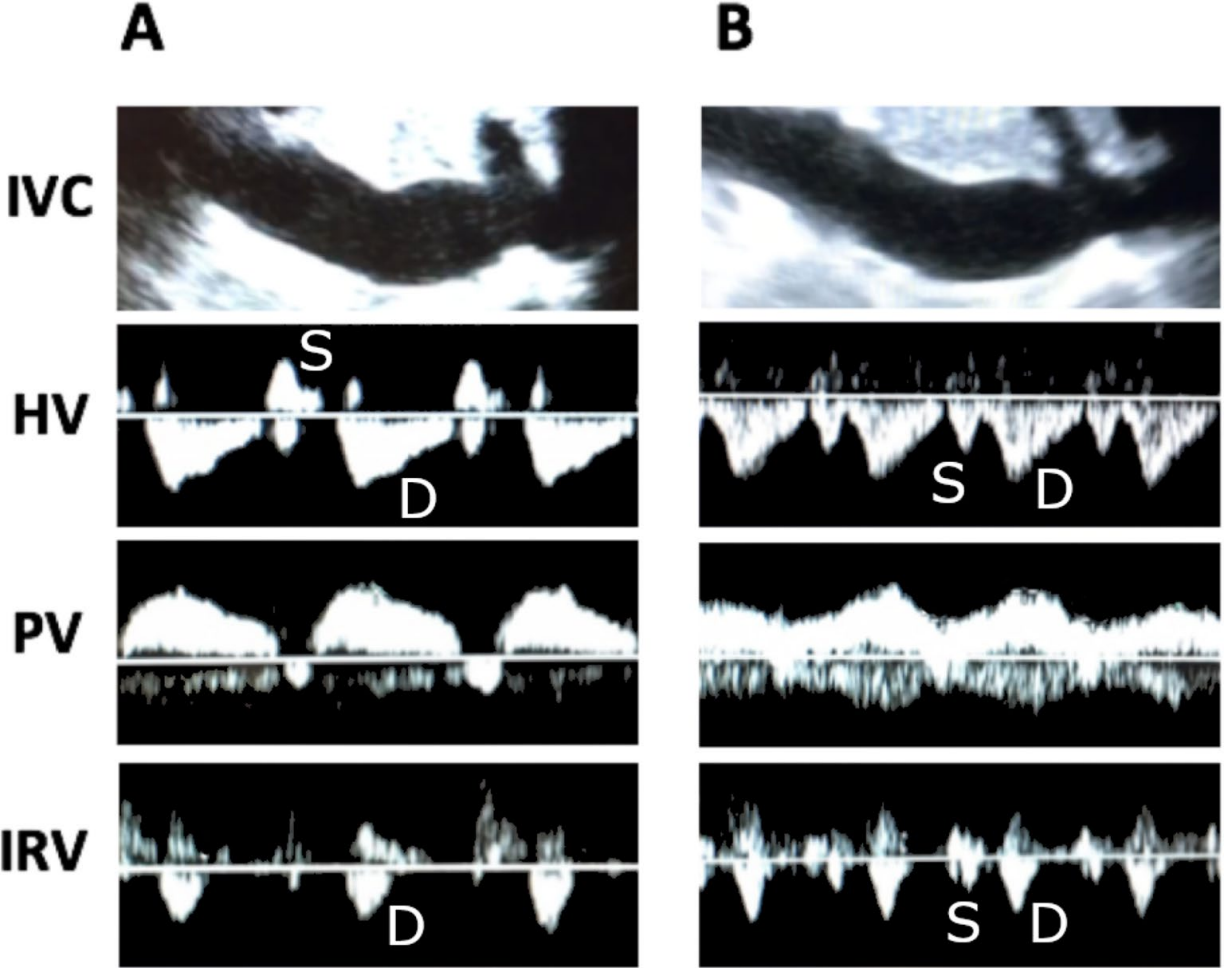
Case 4: **a** initial congestive Doppler profiles. **b** Doppler profiles after hemodialysis with ultrafiltration of 2.5 L

Three weeks later the patient presented to the ED for worsening lower extremity edema and ascites. Physical exam was notable for Grade II ascites and lower extremity edema. Vital signs were HR 52, BP 94/62 mmHg, and O2 saturation of 98%. The creatinine was 311 mmol/L (3.52 mg/dL) and the potassium was 6.7 meq/L. VExUS assessment showed an IVC of 30 mm with no inspiratory collapse, hepatic vein with systolic flow reversal, a portal vein with greater than 100% pulsatility, and discontinuous monophasic intrarenal venous flow (Fig. 5a). POCUS showed normal LV systolic function, a dilated RV, and severe tricuspid regurgitation. B-type natriuretic peptide (BNP) was 1404 pg/mL. Given the severe venous congestion and hyperkalemia, the patient was treated with furosemide 200 mg IV. However, despite this treatment the patient produced no urine. A high-flow catheter was placed and hemodialysis was started with ultrafiltration of 2.5 L. Immediately after ultrafiltration, VExUS scan was repeated showing improvement in venous congestion. The IVC was persistently plethoric (30 mm), the hepatic vein improving to S < D, the portal vein pulsatility decreasing to 45%, and intrarenal venous Doppler became an interrupted biphasic pattern (Fig. 5b). Moments later the patient spontaneously produced 800 mL of urine. A furosemide infusion (200 mg/day) + spironolactone 50 mg BID was started and eventually achieved a negative fluid balance of 15.5 L. Serum creatinine improved to 151 mmol/L (1.71 mg/dL) and the patient was discharged home with oral bumetanide and spironolactone.

This case again demonstrates the value of VExUS in differentiating inadequate from excessive diuretic therapy. Initially, because of the rise in creatinine, the patient was mistakenly determined (without VExUS exam) to be over aggressively diuresed. Cessation of diuretic therapy then led to further renal dysfunction and clinical decompensation. The use of VExUS not only implicated venous congestion as the major culprit in the patient’s deterioration, but served as a guide to both the initiation of ultrafiltration dialysis when furosemide failed, and served to guide titration of the ultrafiltration therapy.

### Case 5

A 28-year-old gravida 5, para 2 female at 32 weeks of a twin pregnancy presented with vaginal bleeding and contractions. The patient had an emergent C-section performed and went into disseminated intravascular coagulation (DIC) requiring massive blood transfusion protocol (> 10 Units). On arrival to the ICU the patient was anuric, hypotensive and on high doses of phenylephrine, norepinephrine, and vasopressin. Her lactate was 11.3mMol/L and Hgb was 6.7 g/dL. The patient’s therapy was being partially guided by a continuous cardiac output monitor which showed a stroke volume variation of 23%, so she also received over 6 L of IV fluid, as she was considered “volume responsive”.

On physical exam the patient was intubated tachycardic with SpO2 of 94% on 80% FiO2 and PEEP of 10 cm H2O.

**Fig. 6:**
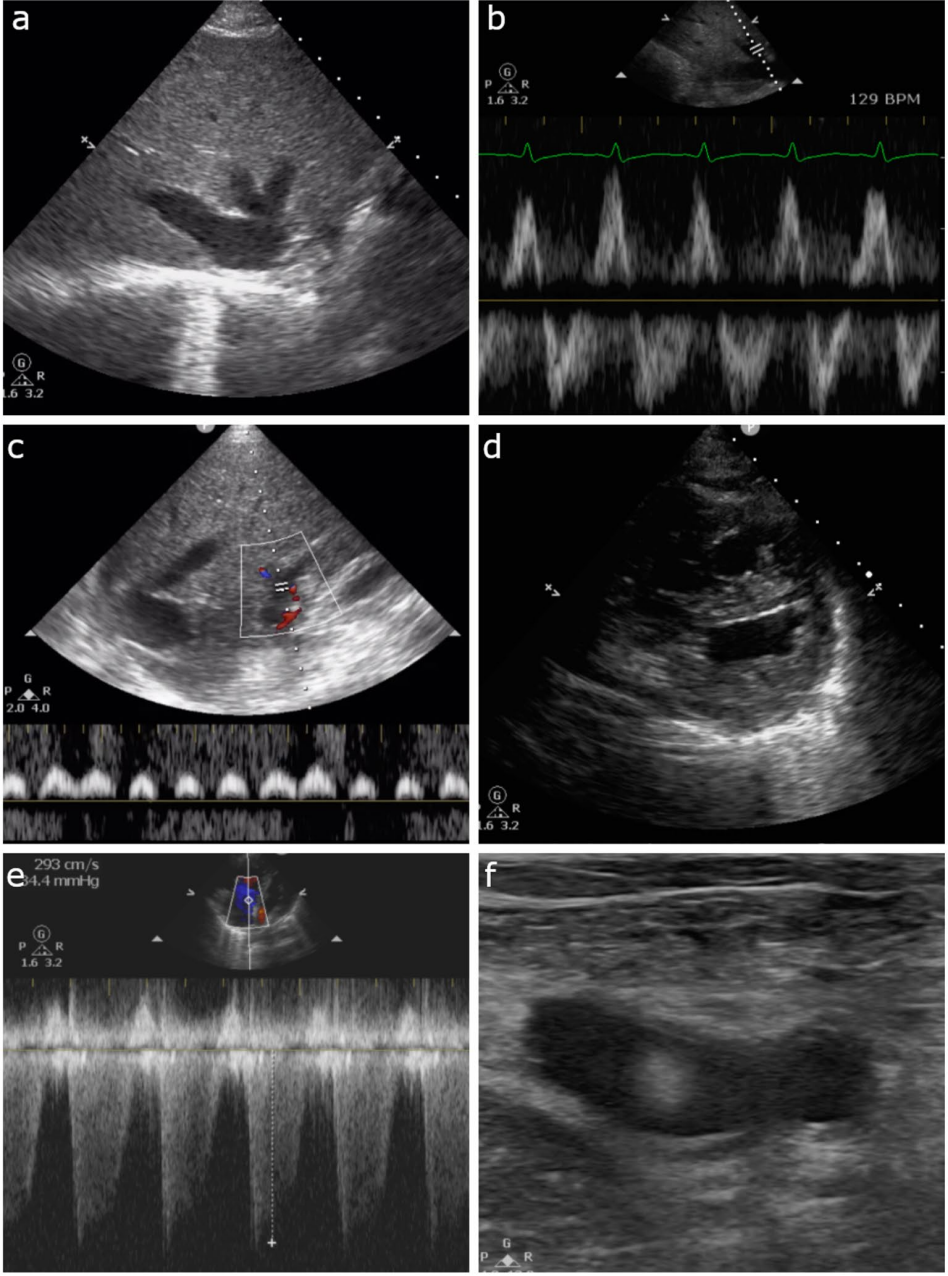
Case 5: **a** dilated IVC. **b** Hepatic vein Doppler with retrograde S wave. **c** Portal vein Doppler with 100% pulsatility. **d** PSAX view of the heart with D-shaped left-ventricle. **e** CW Doppler at the tricuspid valve showing tricuspid regurgitation. **d** Mobile clot seen in the left femoral vein

POCUS showed a dilated IVC, a retrograde S wave on hepatic Doppler, and 100% pulsatile portal vein. In addition, the cardiac ultrasound showed an enlarged right ventricle with a D-sign shape and severe tricuspid regurgitation. The patient had diffuse B-lines in both lung fields and on lower extremity ultrasound, a mobile clot was seen in the left common femoral vein (Fig. 6a–f). The overall VExUS pattern was Grade 3 signifying severe venous congestion.

A CT pulmonary angiogram was obtained showing bilateral small subsegmental pulmonary emboli. Since the patient was anuric, nephrology was consulted for emergent ultrafiltration and 5 L of fluid was taken off within 24 h. Anticoagulation was contraindicated because of the recent C-section and active DIC. An IVC filter was placed for the mobile DVT.

Over the next 2 days the patient’s lactate normalized and vasopressor requirements improved. The patient was eventually extubated 3 days later and discharged after 17 days total of hospitalization.

In this case, the use of VExUS demonstrated that the patient's refractory shocked state was related to volume overload as well as the already recognized RV dysfunction. Without the use of VExUS the RV dysfunction may have been only attributed to the pulmonary emboli.

The continuous cardiac output monitor provided the clinicians erroneous data with regard to the patient’s volume status, likely due to the severe RV dysfunction and subsequent interventricular dependence hemodynamic factors.

The use of VExUS demonstrated significant venous congestion and led to an active fluid offloading strategy rather than continued attempts to use ventricular preloading to resolve the hypotension and shock. This strategy was further supported by the echocardiographic “D-sign” showing that the RV dilation from volume overload was encroaching on LV diastolic filling. With removal of the excess volume, the dysfunctional septal shift resolved, thus increasing left ventricular filling and cardiac output.

## Venous congestion and the VExUS grading system: pathological basis, pitfalls and limitations

**Fig. 7:**
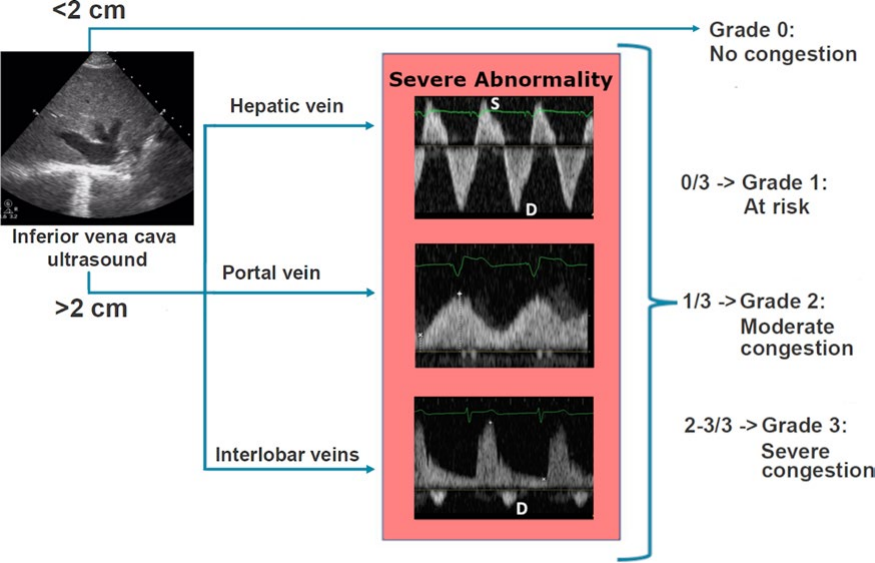
Doppler profiles showing severe congestive patterns

Systemic venous congestion can be evaluated and quantified by Doppler interrogation of multiple splanchnic organs. The VExUS grading system utilizes Doppler evaluation of the hepatic vein, portal vein and intrarenal venous vein. In the presence of a plethoric inferior vena cava, each of these veins are evaluated and assigned to either being normal, mild congestion, or severe congestion. VExUS grade 0 is with no sign of congestion in any organ, VExUS grade 1 is with only mild congestive findings, VExUS grade 2 is with severe findings in only one organ, and VExUS grade 3 is with severe congestive findings in at least 2 of 3 organ systems (Fig. 7).

Hepatic vein Doppler patterns are the result of interactions between venous return-driven by the mean systemic filling pressure (Pmsf) and the right atria and right ventricle. Pmsf is generated by the elastic recoil of the sum total of the venous system. The venous system is highly compliant and acts as a large blood reservoir that can be recruited in times of hypovolemia via an increase in venous tone. Blood volume that occupies the venous compartment without distending the walls of the veins is called unstressed volume, and exerts no influence on venous return. However, any additional volume that distends the walls of the venous system will generate a pressure due to elastic recoil of the veins. The pressure resulting from this recoil, known as the Pmsf, serves as the driving force that generates venous return to the right ventricle. Forces impeding venous return are the resistance of the venous system and the right atrial pressure that exists due to imperfect efficiency of the right ventricle. During the cardiac cycle, when right atrial pressure exceeds Pmfs, the blood flow will be away from the heart and conversely, flow will be towards the heart when Pmfs exceeds right atrial pressure. Therefore, normal hepatic Doppler waveforms are characterized by an initial above the baseline wave associated with right atrial contraction, followed by below the baseline flow during systole (due to right atrial relaxation, movement of the tricuspid annulus toward the apex of the heart, and an increase in atrial volume capacity as the ventricles decrease their volume within the non-compliant pericardium), and finally a below the baseline flow wave during diastole as the right atrium becomes a passive conduit for blood to enter the relaxing right ventricle. These waves are known as the A, S, and D waves, respectively, and correspond to the well-known A wave, X descent, and Y descent of the jugular venous pulse. Generally, as average right atrial pressures rise, the A wave becomes more prominent and the S wave will decrease in amplitude relative to the D wave until there is a systolic reversal of flow and fusion with the A wave.

Under normal circumstances, the venous compartment is highly compliant with high capacitance, and therefore, with increasing distance from the heart, the venous pulse is dampened so that in the smaller veins, flow becomes undulating and phasic in nature. However, in states of right ventricular failure or intravascular volume overload, the venous compartment becomes congested and the limits of venous compliance are reached. Under these circumstances, the normal dampening of the venous pulse due to the compliant nature of the smaller veins is lost, and the pulsations are transmitted back into the smaller veins.

The portal vein is separated from the rest of the systemic venous circulation by the hepatic sinusoids that it empties into. The hepatic sinusoids then drain into the hepatic veins that finally drain into the IVC. Due to this distance from the heart, normal portal venous flow is undulating and phasic in nature and blood travels in a hepatopetal direction at about 20 cm/s. As venous congestion rises, the hepatic vein’s retrograde flow generated by atrial contraction is transmitted up through the hepatic sinusoids and into the portal vein where it gives rise to impedance to the portal hepatopetal flow. This causes the normal undulating flow to become progressively pulsatile and this phenomenon is compounded when there is systolic reversal of venous return to the heart. Eventually, the pulsatility becomes significant enough to cause a biphasic or back-and-forth pattern of portal flow.

Intrarenal venous Doppler waveform obtained from the interlobar or arcuate vessels is usually considered adequate when both arterial (above the baseline) and venous (below the baseline) flow signals are seen clearly for two or more cardiac cycles.

The intrarenal venous Doppler signal is normally a continuous monophasic flow below the baseline. With increasing venous congestion, the venous flow becomes pulsatile, then progresses to an interrupted biphasic flow which correlates to the S and the D waves of the hepatic vein’s flow.

Similar to the hepatic vein pattern, as venous congestion further worsens, the S wave becomes smaller and the D wave is more pronounced until the S wave disappears entirely, leaving only a monophasic D wave.

The intrarenal venous Doppler waveform is considered abnormal when there is discontinuous venous flow with either a systolic/diastolic (discontinuous biphasic) pattern or a diastolic only pattern (discontinuous monophasic).

The assessments presented in this case series are not without important caveats. The IVC can be compressed by increased intra-abdominal pressure independently of right atrial pressure [8]. In chronic pulmonary hypertension, it can experience significant size remodeling [9]. The hepatic vein might not show significant alterations even in severe tricuspid regurgitation if right atrial compliance is preserved [10]. The portal vein can display a pulsatile flow in thin healthy subjects without venous congestion [11], this is also the case in patients with arteriovenous malformations [12]. In patients with stiff liver parenchyma as in cirrhosis or non-alcoholic fatty liver disease, transmission of pressure from the right atrium through the liver sinusoid is dampened and this may lead to a non-pulsatile portal vein even in the presence of severe venous congestion [13, 14]. The intrarenal venous doppler is technically difficult to obtain which can lead to suboptimal recordings [15]. It is also possible that parenchymal renal disease could alter intre-renal doppler venous waveforms [15]. Given these limitations, it is important to avoid interpretation of any of these exams in isolation. Performing a protocolized exam including IVC, HV, PV and IRVD will increase the overall accuracy of the evaluation. In support of this idea, the protocolized combination of scans, summarized by the VExUS grading system, showed increased predictive value for AKI in cardiac surgery patients [7].

## Discussion

These cases illustrate how assessing venous splanchnic congestion can be of critical importance in a wide variety of clinical scenarios. The vague and somewhat unclear concept of “volume status” remains a challenge for clinicians in general due to the limitations of physical examination. Indeed, in many patients, identifying the presence of venous congestion associated with potential end-organ dysfunction may not be possible without invasive monitoring or ultrasound assessments. But beyond the definition of euvolemia, the more practical questions center around when to give or when to remove fluid. While VExUS will not likely provide much information on the need for fluid, it may provide stop points to fluid resuscitation and identify patients who are likely to tolerate and benefit from fluid removal. While the focus in fluid resuscitation has long been cardiac output or forward flow, the literature suggests strongly that venous congestion, as determined by CVP or by venous Doppler indices, will at a certain point offset the benefits of increasing said forward flow [4–7, 16]. In fact, Vellinga et al., in studying the microcirculation, showed that a CVP above 12 actually resulted in a decreased tissue perfusion, and was associated with sluggish capillary flow which they termed “microcirculatory tamponade” [17].

This knowledge should prompt the clinician to be keenly aware of the double-edged nature of fluids and to use available tools at the bedside to tailor fluid resuscitation or removal to avoid pathological markers of venous congestion.

## Conclusion

As time passes, positive experiences of first-line clinicians using venous congestion indices as a guide to the management of intravascular volume accumulates in several different clinical settings. While there remains a lack of properly controlled interventional studies regarding the effectiveness of a strategy based on such an approach, there are good reasons to believe that using an approach incorporating POCUS would have clear advantages compared to current practice characterized by high variability among healthcare providers. The VExUS as a window on the venous pathophysiology, might be a key to achieving precise fluid management.

The rapid evolution of knowledge about the VExUS assessment should be of interest to all first-line clinicians involved in daily decision-making about fluid balance management. Moreover, it adds to the growing body of evidence that a modern clinician should be proficient in POCUS to ensure optimal care to their patients.

## Data Availability

The datasets used and/or analyzed during the current study are available from the corresponding author on reasonable request. Video material illustrating VExUS scanning and grading can be found here: https://thinkingcriticalcare.com/2020/02/28/a-vexus-mini-tutorial-foamed-pocus/. https://westernsono.ca/screencasts/miscellaneous/solid-organ-doppler-of-venous-congestion/.
